# Type 2 Diabetes Mellitus and the Risk of Hepatitis C Virus Infection: A systematic review

**DOI:** 10.1038/srep02981

**Published:** 2013-10-18

**Authors:** Xuan Guo, Min Jin, Ming Yang, Ke Liu, Jun-wen Li

**Affiliations:** 1 Institute of Health and Environmental Medicine; Key Laboratory of Risk Assessment and Control for Environment & Food Safety, No. 1, Dali Road, Tianjin, 300050, China; 2 Department of Geriatrics, West China Hospital of Sichuan University, No. 37, Guoxue Alley, Chengdu, Sichuan, 610041, China; 3 Department of Occupational Health & Radiological Health; Zigong Center for Disease Control and Prevention, No. 1296, Huichuan Road Zigong, Sichuan 643000, China

## Abstract

The aim of this study was to evaluate the relationship between type 2 diabetes mellitus (T2DM) and hepatitis C virus (HCV) infection and to examine whether T2DM enhances the risk of HCV infection compared with the risk in the general population. We followed standard guidelines to perform a meta-analysis. The associated literature was selected based on the established inclusion criteria. The summary odds ratio (OR) and 95% confidence interval (95% CI) were used to investigate the strength of the association. Through electronic database and manual searching, 22 studies were identified for the final analysis, which included a total of 78,051 individuals. Based on the random effects model, the meta-analysis results showed that patients with T2DM were at a higher risk of acquiring HCV infection than non-T2DM patients (summary *OR* = 3.50, 95% *CI* = 2.54–4.82, *I*^2^ = 82.3%). Based on the current limited evidence, this study suggests that T2DM is associated with increased susceptibility to HCV infection.

Hepatitis C virus (HCV) infection and type 2 diabetes mellitus (T2DM) are two major public health problems associated with increasing complications and mortality rates worldwide^1^,^2^. HCV infection is a cause of chronic liver disease, cirrhosis, and hepatocellular carcinoma (HCC) in Western countries and affects an estimated total of 170 million individuals worldwide^3^,^4^. Meanwhile, Data reported by the World Health Organization (WHO) in 2000 showed that the estimated prevalence of T2DM is approximately 2.8% among adults aged over 20 years^5^. Both diseases present a large health care burden. Moreover, HCV infection and T2DM may coexist in an individual^6^.

The development of HCV infection is a multi-factorial process associated with a variety of risk factors, as observed with all other infectious diseases^7^. The major risk factors associated with the development of infection include virus-related factors (e.g., viral load or genotype) and host-related factors, such as age, gender, alcohol consumption, blood transfusion status, obesity, immune status, and co-infections^8^. One important cofactor is T2DM. T2DM has been shown to modify the course of hepatitis C, even at the insulin resistance (IR) stage, which precedes the development of overt diabetes^9^,^10^. And it is now widely recognized that chronic hepatitis C is a metabolic disease that is strongly associated with T2DM and IR^11^.

In recent studies, however, several researchers have focused on the seroprevalence of viral hepatitis markers in individuals who suffer from diabetes. They reported an excessive prevalence of HCV infection among patients with T2DM compared with the general population, which was considered to be the result of more frequent exposure to medical interventions and instrumentation and compromised immunity, leading to an increased risk of HCV infection^12^,^13^,^14^. The association between T2DM and liver disease was recognized over 30 years ago^15^. Since then, numerous observational studies assessing the association between HCV and T2DM have been published, which provided heterogeneous results such as positive association^13^,^16^,^17^ no significant association^18^ and even negative association^19^,^20^. This result may be partly due to the differences in the sources of controls, case definitions, sample sizes, and underlying target populations. Several general reviews on the association between HCV infection and DM have been published^3^,^7^,^21^,^22^,^23^; however, they have typically been limited in scope or by non-systematic reasoning. Currently, only meta-analyses demonstrating that HCV infection can promote the increased prevalence of T2DM have been reported; however, whether T2DM can increase the prevalence of HCV infection still need to be investigated^24^,^25^.

Based on the literature, we hypothesized that individuals with T2DM are more prone to HCV infection. The objectives of the present study were, therefore, (1) to overcome the limitations of previous studies in being underpowered by performing an evidence-based quantitative meta-analysis of the published data to assess whether T2DM conveys an excess risk of contracting HCV infection compared with that observed among the general population; and (2) to quantify and appropriately qualify any observed excess risk to identify any high-risk subgroups and explore potential sources of between-study heterogeneity.

## Results

### Literature search and meta-analysis database

**Figure 1:**
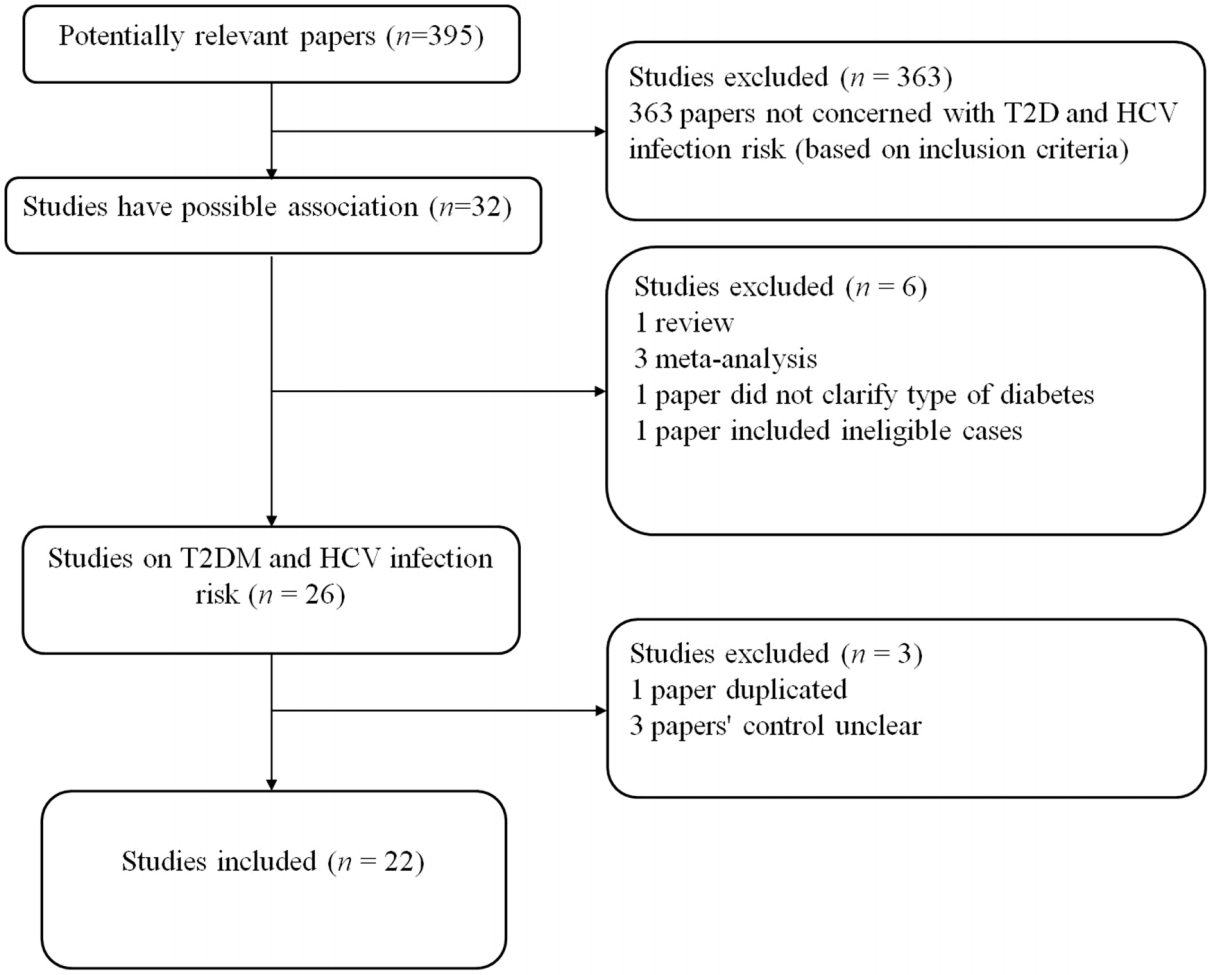
Flow diagram of included/excluded studies.

A total of 395 studies on T2DM with respect to HCV infection were identified and screened for inclusion in the study. Of these studies, 363 were found to be irrelevant and were excluded; 32 were finally selected. Then, 3 studies were excluded because they were meta-analyses^24^,^25^,^26^, 1 study was excluded because it was a review^27^, 1 study was excluded because it included ineligible cases^28^, and 1 study was excluded because it did not clarify the type of diabetes^29^. An additional 3 studies were excluded because the distribution of the control groups was unclear^30^,^31^,^32^. Moreover, 2 study samples may have been duplicated in both the case and control groups, and the study with the greater number of cases was selected^8^,^33^. In total, 22 case-control studies were selected (Supplementary Table 2, Figure 1).

### Assessing the quality of each case-control study

The Newcastle-Ottawa Scale (NOS) was applied for the quality assessment of each study, and each study was judged based on three broad perspectives: the selection of the study groups, the comparability of the groups, and the ascertainment of the exposure^34^. A maximum of 1 star was awarded for each numbered item within the selection and exposure categories, and two stars were awarded for comparability. A study that acquired more stars was considered to be of higher quality. The studies included in our meta-analysis had 6 or more stars (Supplementary Table 3).

### Test of heterogeneity

**Figure 2:**
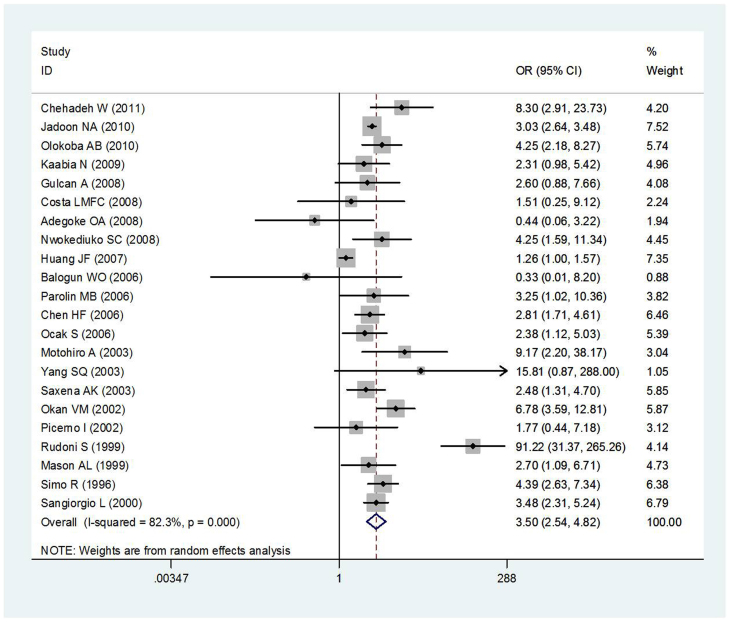
Meta-analysis of the association between T2DM and HCV infection risk.

Twenty-two studies discussed the relationship between T2DM and HCV infections for a total of 12,426 cases and 65,625 controls. Ten of those studies were conducted in Asia (6 in West Asia and 4 in East Asia), 5 were conducted in Africa, 4 were conducted in Europe, 2 were conducted in Latin America, and 1 was conducted in North America. Half of the studies utilized hospital control populations (11 out of 22). Figure 2 show, a large between-study heterogeneity was observed for all studies (*Q* = 118.94, *P* < 0.001, *I*^2^ = 82.3%).

### Quantitative data synthesis

**Figure 3:**
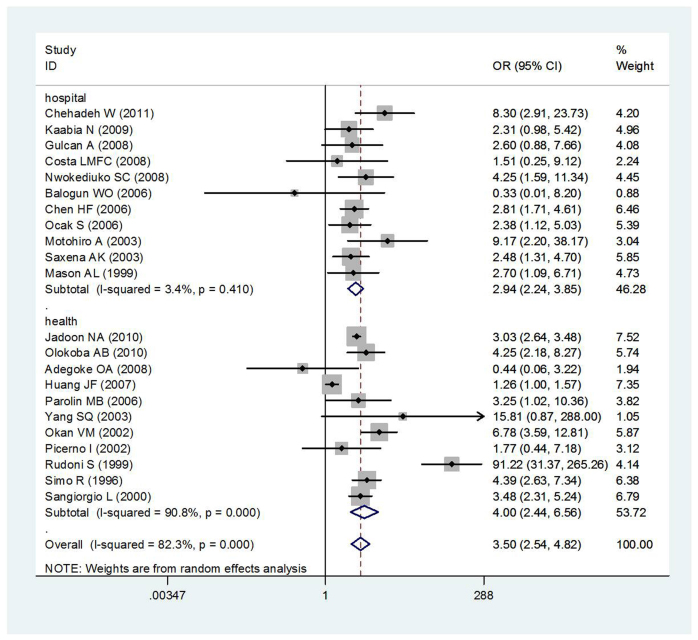
Meta-analysis of the association between T2DM and HCV infection risk according to control source.

**Figure 4:**
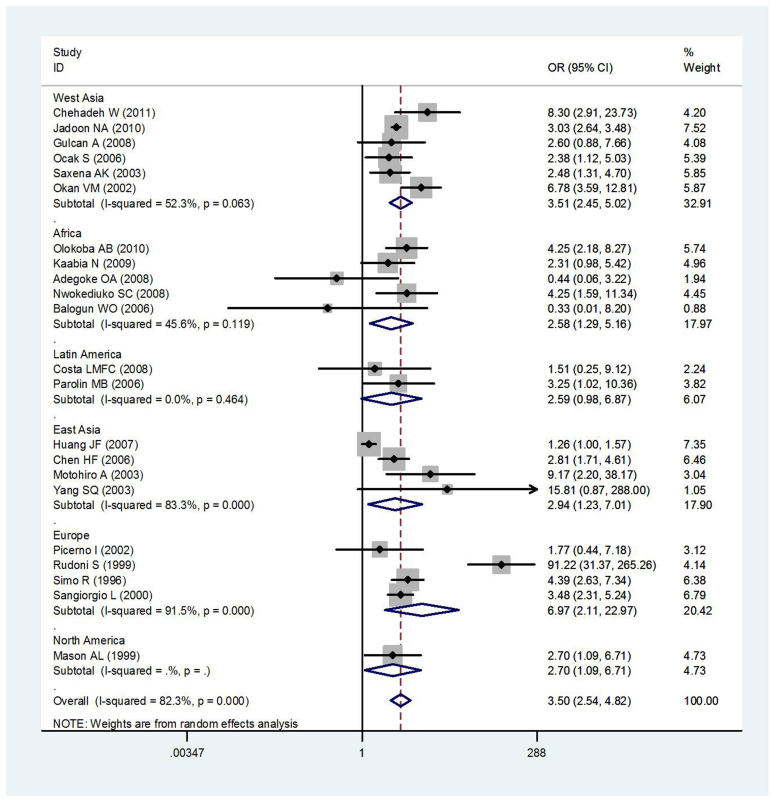
Meta-analysis of the association between T2DM and HCV infection risk according to geographic region.

Area difference and stratification of control sources did not resolve the observed heterogeneity (Figures 3 and 4). The summary OR between T2DM and HCV infection risk was 3.50 (95% *CI*: 2.54–4.82) based on the random effects model. There was, however, substantial heterogeneity among studies (*I^2^* = 82.3%, heterogeneity *P* = 0.000).

### Subgroup analysis

Area-specific analyses revealed low between-study heterogeneity for Africa (*OR* = 2.58, 95% *CI* = 1.29–5.16, *I^2^* = 45.6%, heterogeneity *P* = 0.119) and Latin America (*OR* = 2.59, 95% *CI* = 0.98–6.87, *I^2^* = 0.0%, heterogeneity *P* = 0.464). Half of studies used hospital control populations and showed low between-study heterogeneity(*OR* = 2.94, 95% *CI* = 2.24–3.85, *I^2^* = 3.4%, heterogeneity *P* = 0.410). In all studies, between-study heterogeneity was observed (*P* = 0.000, *I*^2^ = 82.3%), and control source stratification did not resolve this heterogeneity (Figure 3).

### Sensitivity analysis

**Figure 5:**
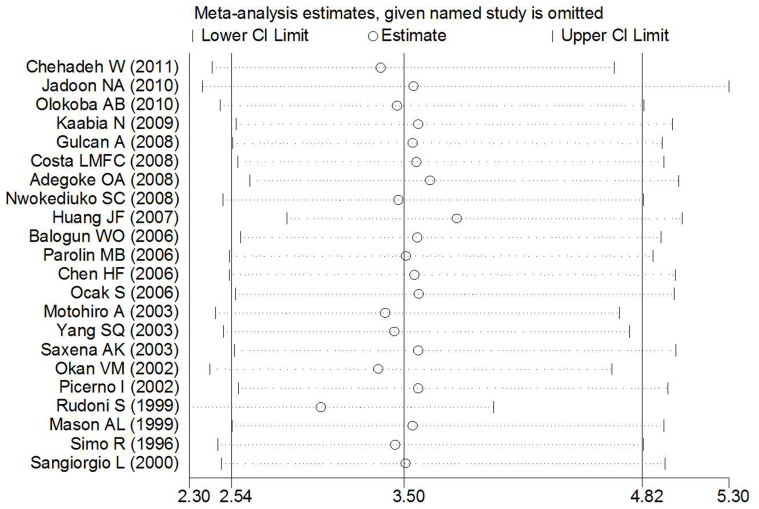
Sensitivity analysis of the meta-analysis of the association between T2DM and HCV infection risk.

One-way sensitivity analysis was conducted to evaluate the stability of the meta-analysis (Figure 5). When any single study was omitted, the statistical significance of the overall effect remained unchanged, thereby suggesting that the data included in this meta-analysis were stable and reliable.

### Bias diagnostics

**Figure 6:**
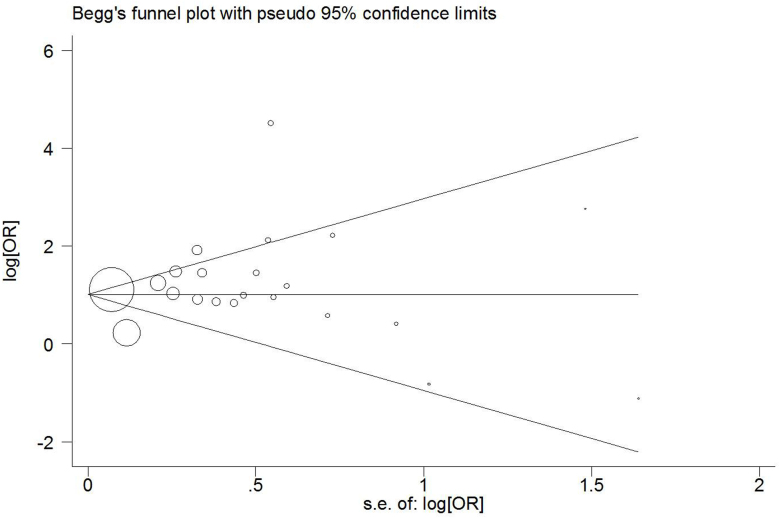
Begg's funnel plot of the meta-analysis of T2DM and HCV infection risk.

Funnel plots were created to assess possible publication biases. As shown in Figure 6, the funnel plots seemed to be very symmetrical. Thus, Egger's linear regression test was applied to quantitatively evaluate the symmetry of the meta-analysis funnel plot (Figure 6). The 95% CI of the intercept included a value of 0 in Egger's publication bias plot, thereby suggesting that the results of this meta-analysis are relatively stable and that publication biases may not exert an evident influence on the results of the meta-analysis.

## Discussion

It is important to note that this is the meta-analysis to specifically examine the association between T2DM and the risk of acquiring HCV in the general population. Among the 22 eligible studies identified for this study, all studies evaluated HCV risk in T2DM cases compared with controls (non-T2DM cases). Our meta-analysis, which combined the ORs of these retrospective studies, demonstrates an approximately 3.5-fold increase in HCV infection risk among T2DM patients. Although there was evidence of potential small study or publication bias among these retrospective studies, this effect appears to be largely explainable by the removal of the single largest study, which did not change the overall trend. And our data indicate that titers of cell secretory virus display a change in gradient with increasing doses of insulin (data not published). Taken together, the findings of our meta-analysis clearly indicate that T2DM was significantly associated with the risk of HCV infection compared with the risk in non-T2DM controls.

In 2008, a meta-analysis of observational studies reported an excessive T2DM risk associated with HCV infection. Specifically, pooled estimators indicated significant DM risk in HCV-infected cases compared with non-infected controls in both retrospective (adjusted OR = 1.68, 95% CI 1.15–2.20) and prospective (adjusted HR = 1.67, 95% CI 1.28–2.06) studies^25^. Meanwhile, a large number of observational studies in which the prevalence of HCV infection among patients with T2DM was assessed have been conducted. For example, a large-scale epidemiological study demonstrated that subjects with T2DM exhibited a higher prevalence of HCV infection (OR = 1.26, 95% CI 1.00–1.57) than non-T2DM controls^35^. In another study, Simo, R. and his coworkers found that patients with T2DM were almost five times more likely to be tested HCV antibodies positive than controls^36^. The course of HCV infection is often completely asymptomatic or displays only a modest increase in serum transaminases; thus, this infection may remain unobserved. If the importance of testing diabetic patients for hepatitis markers is uncontested, as supported by the above findings, it may be possible to diagnose and prevent HCV infection early.

The association between chronic HCV infection and T2DM seems biological plausibility, but it remains to be determined whether HCV infection can lead to diabetes or vice versa. Some data regarding the prevalence of antibodies in HCV patients with diabetes and in T2DM patients with HCV are conflicting. Some potential bias may occur in clinic-based studies that target a specific disease group; therefore, a clear biological link between HCV infection and T2DM has not been demonstrated. The mechanisms by which T2DM may induce chronic HCV infection could be manifold. First, T2DM is a common endocrine disorder encompassing multifactorial pathogenetic mechanisms including IR, increased hepatic glucose production, and a defect in insulin secretion, all of which contribute to the development of hyperglycemia^37^,^38^. It has also been suggested, based on a few in vitro studies, that HCV replication may be favored by hyperinsulinemia and/or the increased serum levels of free fatty acids observed in patients with IR and T2DM^39^,^40^. Additionally, T2DM is, to some extent, associated with an immunocompromised state, which leads to derangements of immune function^41^. Both IR and T2DM may play a role in the alteration of the natural course of HCV infection, thus leading to enhanced steatosis, steatohepatitis, and liver fibrosis^41^,^42^. Moreover, most patients with diabetes mellitus often withdraw blood and perform glycemic assessments at home with the aid of family members. It would be informative for diabetic patients to perform an anamnesis focused on the eventual risk factors associated with viral hepatitis (e.g., transfusion, hospitalization, and eventual surgical operations) and other factors that may induce an eventual alteration of hepatic function (e.g., alcohol abuse and hemochromatosis).

There are some limitations to our meta-analysis. First, the majority of the studies did not report HCV and T2DM status according to subgroups. Therefore, we were unable to perform stratification using those variables, which may have provided a better explanation for some results. Second, heterogeneity of the studies may have limited the efficacy of our meta-analysis to demonstrate trustworthy associations between HCV infection and T2DM. Third, despite performing stratification by control type and study region, it was less likely to resolve the heterogeneity due to variations of subgroup sample sizes. Furthermore, we did not include any unpublished data, which may lead to over/under estimation and/or bias. The source of publication bias for that particular variant remains unknown. Most, if not all, observational studies have the potential for ascertainment bias, particularly studies in which diabetes status was defined via self-report^43^,^44^. Although confounding factors were addressed in many of these observational studies, it is likely that there may be unmeasured confounding factors that may induce bias. Finally, because consistent data regarding patient level were not available for each study, we were unable to make further inferences regarding important factors such as genotype, which were not included in most of the primary studies.

Despite these limitations, the current meta-analysis has a few advantages and provides insight for our future study. First, no other meta-analyses have been performed regarding this issue. Importantly, this study provided insight into the questions of whether HCV infection is a risk factor for T2DM and whether patients with T2DM have an increased susceptibility to HCV infection. Additionally, the results of this meta-analysis may have important clinical implications. Given the demonstrated increased risk of HCV infection associated with T2DM, a strong case can be made to screen for HCV infection in T2DM individuals. Finally, these data provide better insight regarding the overall burden of disease for chronic hepatitis C. Indeed, if the risk of HCV infection increases with the increased duration of T2DM, it may become a prominent T2DM-induced health problem in some patients. As some reports have shown that the treatment of IR may improve HCV infection, some T2DM patients at high risk for HCV infection may benefit from hypoglycemic agents^45^. Further studies are required to determine whether HCV infection could be prevented or reversed with successful T2DM treatment.

In conclusion, our meta-analysis demonstrates an association between T2DM and HCV infection in adults. Specifically, subjects with T2DM displayed a higher prevalence of HCV infection than non-T2DM controls. The current meta-analysis also reflects the need for more extensive studies that include larger populations.

## Methods

### Literature search strategy to identify relevant studies

We followed published guidelines while conducting and reporting the meta-analysis^46^. A search was performed, without a language limitation, to identify all papers published until August 2012. Searches were performed using the databases CBM, EMBASE, PubMed, and Ovid and publishers such as CNKI, VIP, ISI, EMSCO, Sciencedirect, and SpringerLinker. A combination of the following key words was applied: “diabetes”, “diabetes mellitus”, “diabetes mellitus, type II” or “type II diabetes”, “hepatitis C”, “hepatitis C virus”, “hepatitis”, or “chronic hepatitis”, “risk factor”, “rate”, “case-control”, “cohort”, “clinical trial”, “epidemiology”, and “observational”. Additionally, we searched the Cochrane Database of Systematic Reviews and Cochrane Central Database of Controlled Trials. We also searched related journals individually. We evaluated potentially associated publications by reviewing their titles and abstracts and then procured the most relevant publications for closer examination. The references in the above-selected papers were also screened for possible overlooks in the initial search.

The following criteria were used for the literature selection for the meta-analysis:

1. Studies concerning the association of type II diabetes with Hepatitis C virus infection or studies that utilized an epidemiological study design to conduct a primary or secondary data analysis;
2. Both case and control groups or exposed and unexposed groups must originate from the same geographically and temporally defined underlying population;
3. Case-control studies;
4. At least 1 comparison group with no T2DM patients;
5. Provision of sufficient data to calculate odds ratios (ORs) comparing HCV infection in T2DM patients with that in non-T2DM patients;
6. Minimum sample size of 100, with a minimum of 50 cases and 50 controls.

Accordingly, the following exclusion criteria were also applied:

1. Study design and definition that were unrelated to the objectives of the present study;
2. Essential information missing;
3. Inclusion of children, post-transplant recipients, dialysis patients, pregnant women, or thalassemia or cancer cases;
4. Indistinguishability of T2DM from sub-clinical hyperglycemia or HCV not being excludable from other causes of hepatitis;
5. Duplicated publications.

After performing the search, all papers were reviewed in accordance with the criteria defined above for further analysis.

### Data extraction

From each study, 2 investigators independently recorded the detailed information using piloted forms, and the following information was extracted: first author's name, year of publication, country where the study was conducted. The controls came from the patients in the same hospital was defined as the hospital controls, and the communities of health survey and blood donor were defined as the health controls.

### Statistical analysis

The association between T2DM and the risk of HCV infection was assessed by calculating ORs with 95% confidence intervals CIs, which were calculated according to Woolf's method^47^. The between-study heterogeneity was determined by performing the *χ*^2^-based *Q* statistics test, and it was considered significant when *P* < 0.10^48^. The *I*^2^ statistic was used as a confirmatory test for heterogeneity, with *I*^2^ < 25%, 25–50%, and >50% representing a low, moderate, and high degree of heterogeneity, respectively^49^,^50^. To examine between-study heterogeneity, stratification by area and control source was conducted. The random effects model was developed using DerSimonian-Laird's method, which assumed that studies were based on populations with varying effect sizes. The study weights associated with both in-study and between-study variances were calculated, considering the extent of variation or heterogeneity^51^. The fixed effects model was developed using Mantel-Haenszel's method, which assumed that studies were sampled from populations with the same effect size, with adjustments made to the study weights according to the in-study variance^52^. An asymmetric plot suggested possible publication bias. Furthermore, funnel plot asymmetry was assessed via Egger's linear regression test method^53^. All of the statistical tests were performed using STATA SE (version 11) software (StataCorp LP, College Station, TX, USA). The methods and findings o f the present review have been reported based on the preferred reporting items for systematic reviews and meta-analysis checklist (PRISMA) (Supplementary Table1)^54^.

## Supplementary Material

Supplementary Information Supplementary Table 1
